# Gastroprotective effects of diosgenin against HCl/ethanol-induced gastric mucosal injury through suppression of NF-κβ and myeloperoxidase activities

**DOI:** 10.1515/biol-2021-0075

**Published:** 2021-07-16

**Authors:** Hengfang Zhao, Xiaoyan Zhang, Bojing Zhang, Xiaoyuan Qu

**Affiliations:** Department of Gastroenterology, Xi’an No. 3 Hospital, The Affiliated Hospital of Northwest University, Xi’an, Shaanxi 710018, P.R. China; Department of Critical Care Medicine, Shaanxi Provincial Hospital of Traditional Chinese Medicine, Xi’an, Shaanxi Province, 710003, China

**Keywords:** antioxidant, anti-inflammatory, gastric injury, myeloperoxidase, nitric oxide

## Abstract

Gastric mucosal injury is caused by an imbalance between the mucosal defense and gastro-irritants, leading to gastroenteritis. Diosgenin is a steroidal sapogenin found in the wild Yam plant that has been reported with several pharmacological properties. The aim of this study is to explore the gastroprotective role of diosgenin on gastric mucosal damage caused by HCl/ethanol in rats. Male Sprague-Dawley rats were intragastrically administered with diosgenin (20 mg/kg) before HCl/ethanol (0.15 M HCl in 98 % ethanol) administration. Omeprazole was used as a positive control. Diosgenin-attenuated oxidative stress by enhancing (*p* < 0.05) antioxidant enzymes, reducing lipid peroxidation (MDA), and modulating nitric oxide (NO) levels. Anti-inflammatory effects of diosgenin were observed by a reduction in pro-inflammatory cytokines (*p* < 0.05), decreased myeloperoxidase (MPO) activities (*p* < 0.05), and histopathological observation of gastric mucosal damage. Western blot analysis provided evidence on the downregulation of NF-κβ by diosgenin. The findings showed that diosgenin has a significant protective role on gastric injury caused by HCl/ethanol, through its antioxidant, anti-inflammatory role, and suppression of NF-κβ and MPO activities.

## 1 Introduction

Gastric injury or gastroenteritis is a common disease among millions of people around the world [1]. Gastric mucosal lining is linked to various exogenous and endogenous protection and maintenance mechanisms such as alkaline mucus secretion, gastric microcirculation, renin–angiotensin system, antioxidant, and enzymatic barriers against gastro-irritants and toxic substances [2]. The gastric mucosa is vulnerable to toxins excreted by *Helicobacter pylori*, noxious matters, alcohol/ethanol, nonsteroidal anti-inflammatory drugs (NSAIDs), hydrochloric acid (HCl), bile acids, and pepsin [3]. Alcohol is one of the highly abused substances in the world, which could lead to upper gastrointestinal bleeding and peptic ulcers. An imbalance between the mucosal defense and gastro-irritants/aggressive materials leads to gastric mucosal injury. Untreated conditions could lead to peptic ulcers accompanied by symptoms of abdominal pain, vomiting, nausea, loss of weight, poor appetite, and bloating [4]. Oxidative stress and inflammation are often linked with the progression of gastric mucosal damage at the cellular level. During inadequate defense by antioxidants and antioxidant enzymes to scavenge the excessive reactive oxygen species (ROS) free radicals, gastric mucosal injury is initiated and is prolonged by infiltration of pro-inflammatory cytokines and neutrophils [5,6].

Natural products are known for their therapeutic potentials against various ailments caused by oxidative stress and have been a choice for research and treatment in recent years due to their effectiveness and safety with less or no side effects [7]. Past researchers have demonstrated the gastroprotective ability of plants extracts and natural active constituents from plants against experimental gastric injury in animal models [8]. A preclinical model of gastric mucosal injury is commonly induced using HCl/ethanol since alcohol abuse is one of the leading causes of gastric injury in humans [9]. Diosgenin is a steroidal sapogenin that could be found as a major bioactive compound in tubers of wild Yam plants (*Dioscorea villosa*) and several other plants including *Costus*, *Smilax*, *Dioscorea*, and *Trigonella* [10,11]. Diosgenin has multiple pharmaceutical advantages including anti-inflammatory [12], hepatoprotective [13], anti-hypercholesterolemic [14,15], anti-osteoporotic [16], prevents spinal cord injury [17], prevents testicular damage in diabetic rats [18], attenuates Parkinson’s disease [19], and modulates insulin resistance and anabolic hormones [20]. The anti-hypercholesterolemic studies have reported that diosgenin is safe for consumption and has significant inhibition toward gastrointestinal uptake of dietary cholesterol and also the regulation of bile acids [15,16]. Antioxidant compounds have the tendency to exert gastroprotective effects. Till date, there are no studies on the gastroprotective properties of diosgenin; hence it was selected for this study with relevance to its pharmacological properties. Gastrointestinal tract disorders induced by alcohol consumption are relatively high in common. A study on HCl/ethanol-induced gastric mucosal injury could give a better understanding of the treatment measures for alcoholic gastroenteritis. Therefore, this study aims to investigate the anti-inflammatory and gastroprotective roles of diosgenin on HCl/ethanol-induced gastric mucosal damage in rats.

## 2 Materials and methods

### 2.1 Chemicals

**Figure 1:**
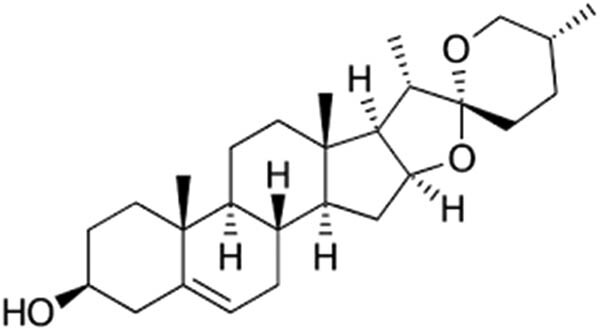
Chemical structure of diosgenin.

Diosgenin, omeprazole, HCl, and ethanol (98%) were procured from Santa Cruz Biotechnology Inc., USA (Figure 1). Chemicals for biochemical analysis, antigens, and antibodies for the ELISA assay were obtained from Sigma, St. Louis, USA, and Abcam, USA. Diosgenin was dissolved in 0.1% tween 80 for experimental administration.

### 2.2 Animals

Male Sprague-Dawley (6–7 weeks) rats (180–200 g) were randomly housed in wire bottom cages for the prevention of coprophagy, in a group of ten (*n* = 10) at 24 ± 2°C temperature, 60% humidity, 12 h light/dark cycle, supplied with standard rodent feed and water.


**Ethical approval:** The research related to animal use has been complied with all the relevant national regulations and institutional policies for the care and use of animals and was approved by the ethical committee of Xi’an Third Hospital (Ethical No.: XATH20200827).

### 2.3 Experimental design and HCl/ethanol-induced gastric injury

All animals were fasted for 24 h and allowed free access to water alone before the initiation of the experiment. The experimental protocol was performed following the methods of Yang et al. [21]. Group I (normal control) was treated with intragastric administration of 0.1% tween 80. Group II served as a gastric model group was administered with HCl/ethanol (0.15 M HCl in 98% ethanol) intragastrically. Group III served as an experimental group treated with diosgenin (20 mg/kg b.w.) by intragastric administration. Group IV served as a positive control treated with omeprazole (20 mg/kg b.w.) by intragastric administration. Groups III and IV received intragastric administration of HCl/ethanol (0.15 M HCl in 98% ethanol), 1 h after the drug administration. The dose of diosgenin was selected based on preliminary studies and past research on the animal model study of diosgenin [18] that was able to prevent oxidative stress; hence, the dosage was modified according to the study of treating gastric mucosal damage in rats [22]. One hour after the administration of HCl/ethanol, the rats were sacrificed through cervical dislocation under deep isoflurane anesthesia, the stomach was dissected longitudinally, and it was washed with ice-cold saline. Consequently, gastric mucosal tissues were subjected to histopathological and biochemical analyses.

### 2.4 Biochemical analysis

The gastric tissues excised from rats were homogenized in cold phosphate-buffered saline (pH 7.4) and centrifuged at 2,000 ×*g* for 10 min at 4°C, and the supernatants obtained were subjected to biochemical assays for reduced glutathione (GSH), catalase (CAT), glutathione peroxidase (GPx), superoxide dismutase (SOD), malondialdehyde (MDA), nitric oxide (NO) levels, and myeloperoxidase (MPO) activity. The remaining aliquots were used for the ELISA assay (Sigma, St. Louis, USA) for interleukin 1-beta (IL-1β), interleukin 6 (IL-6), and tumor necrosis factor-alpha (TNF-α) following the manufacturer’s guidelines. Commercial colorimetric assay kits (Sigma, St. Louis, USA) were used for the determination of CAT, GSH, GPx, SOD, MDA, and NO levels and MPO activity. Protein determination in aliquot was done by Bradford’s assay using bovine serum albumin (BSA) as a standard. Briefly, the decomposition rate of H_2_O_2_ was observed at 240 nm for CAT determination, formation of 5-thiol-2-nitrobenzoic acid at 412 nm was observed for GSH determination, the oxidation rate of NADPH to NADP^+^ was observed at 340 nm for GPx determination, inhibition rate of pyrogallol auto-oxidation was observed at 470 nm for SOD determination, formation of thiobarbituric acid (TBA) conjugates was observed at 535 nm for MDA determination, production of stable NO metabolite with Griess reagent was observed at 540 nm for NO determination, and degradation rate of peroxide was observed at 412 nm for MPO determination.

### 2.5 Histopathological analysis

A portion of the gastric tissue was fixed in formaldehyde (4%) for 24 h. The tissues were then dehydrated and paraffin embedded. Embedded tissues were sliced at 5 µm thickness, deparaffinized, and stained with hematoxylin and eosin (H&E). The samples were imaged under the light microscope. Histopathological scoring was performed according to the study by Li et al. [23] by a pathologist who was not aware of experimental groups, mucosal edema was given a score range of 0–4, loss of epithelial cells was given a score range of 1–3, infiltration of inflammatory cells was given a score range of 1–3, and hemorrhage was given a score range of 1–4.

### 2.6 Western blot

Western blot analysis was performed to evaluate the involvement of the NF-κβ pathway in the gastric mucosal injury induced by HCl/ethanol. Gastric tissues were homogenized using lysis buffer with protease and phosphatase inhibitor cocktail and centrifuged at 4°C for 10 min at 16,000 ×*g*, and the aliquot was used for western blot analysis. Aliquots with equal amounts of protein were separated through electrophoresis on 10% SDS-PAGE and transferred to PVDF membranes. The membranes were blocked with 5% skimmed milk at room temperature for 2 h in Tris-buffered saline with 0.1% Tween 20. Then, the membranes were incubated overnight with primary antibodies against Ikβα (1:1,000 dilutions; ab32518, Abcam, USA), p-Ikβα (1:1,000 dilutions; ab133462, Abcam, USA), p65 NF-κβ (ab16502, Abcam, USA; 1:1,000 dilutions), p-p65 NF-κβ (1:1,000 dilutions; ab86299, Abcam, USA), and glyceraldehyde-3-phosphate dehydrogenase (GAPDH; 1:500 dilutions; ab181602, Abcam, USA). The membranes were washed with TBST and further incubated with anti-rabbit horseradish peroxidase–conjugated antibody (1:5,000 dilutions; ab97051, Abcam, USA) for 1 h. Immunoreactive protein expressions were detected and quantified with the enhanced chemiluminescence system (Bio-Rad Lab version 6.0, USA).

### 2.7 Statistical analysis

All values are displayed as mean ± SEM for 10 rats. Statistical significance was verified with SPSS version 19.0 by performing ANOVA followed by Tukey’s *post hoc* analysis. All *p* < 0.05 were regarded as significant.

## 3 Results

### 3.1 Effects of diosgenin on GSH, GPx, SOD, and CAT activities

**Figure 2:**
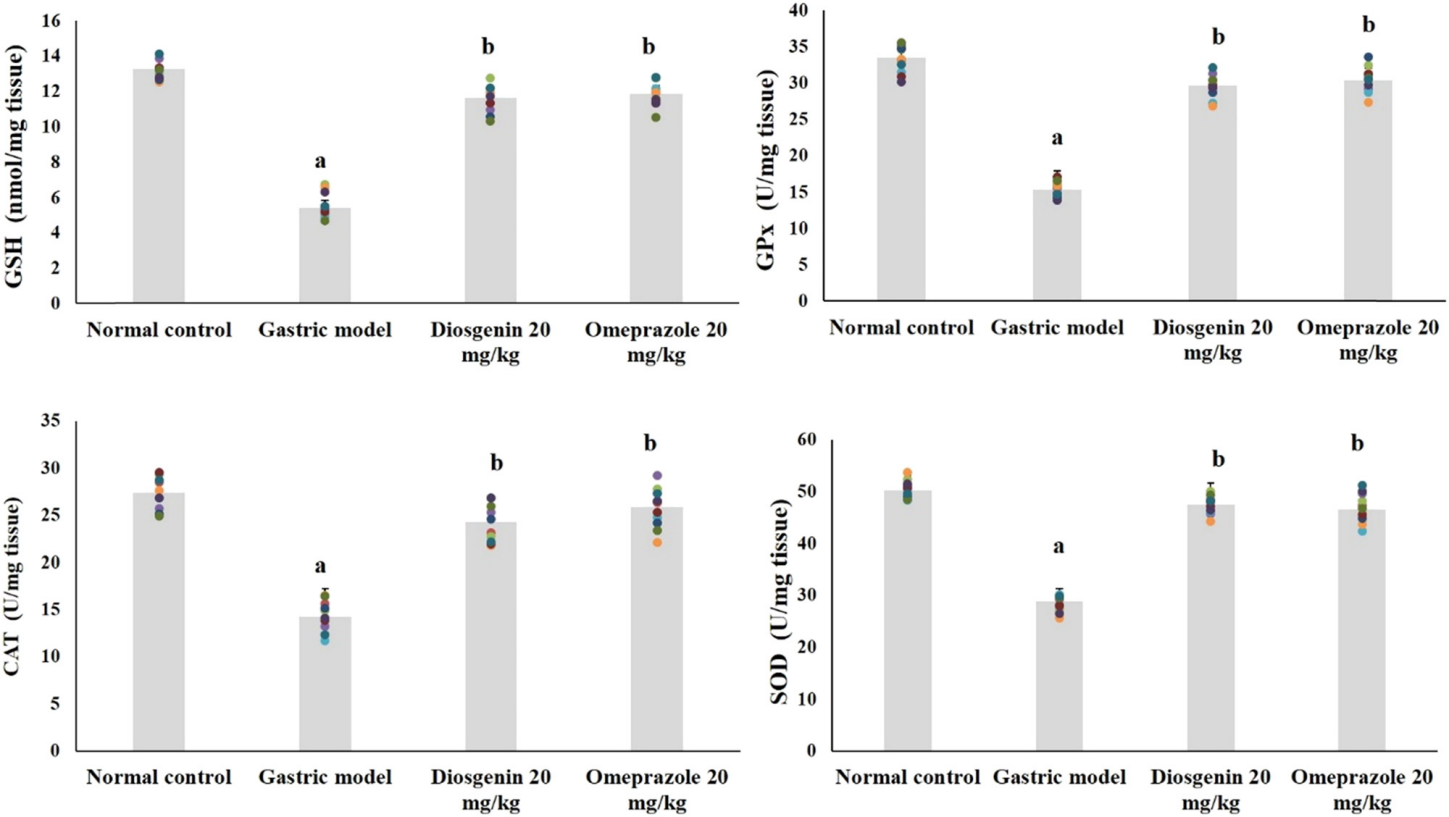
Diosgenin prevents oxidative stress in gastric ulcer-induced rats. Values are expressed as mean ± SEM of antioxidant and antioxidant enzymes in rats (*n* = 10). *p*(a) < 0.05 compared to the normal control. *p*(b) < 0.05 compared to the gastric ulcer model. GSH = reduced glutathione; GPx = glutathione peroxidase; SOD = superoxide dismutase; CAT = catalase.

Antioxidant enzymes and nonenzymatic GSH in the normal and experimental rats are shown in Figure 2. GSH levels were reduced significantly (approximately 59%) in the gastric model group (*p* < 0.05) from the control group. Similarly, levels of antioxidant enzymes were decreased (*p* < 0.05) approximately 54% for GPx, 42.5% for SOD, and 48% for CAT by HCl/ethanol in the gastric model group compared to normal. Compared to the model group, GSH, GPx, SOD, and CAT levels were elevated by the intragastric administration of diosgenin (*p* < 0.05) and by positive control omeprazole.

### 3.2 Effects of diosgenin on MDA and NO levels

**Figure 3:**
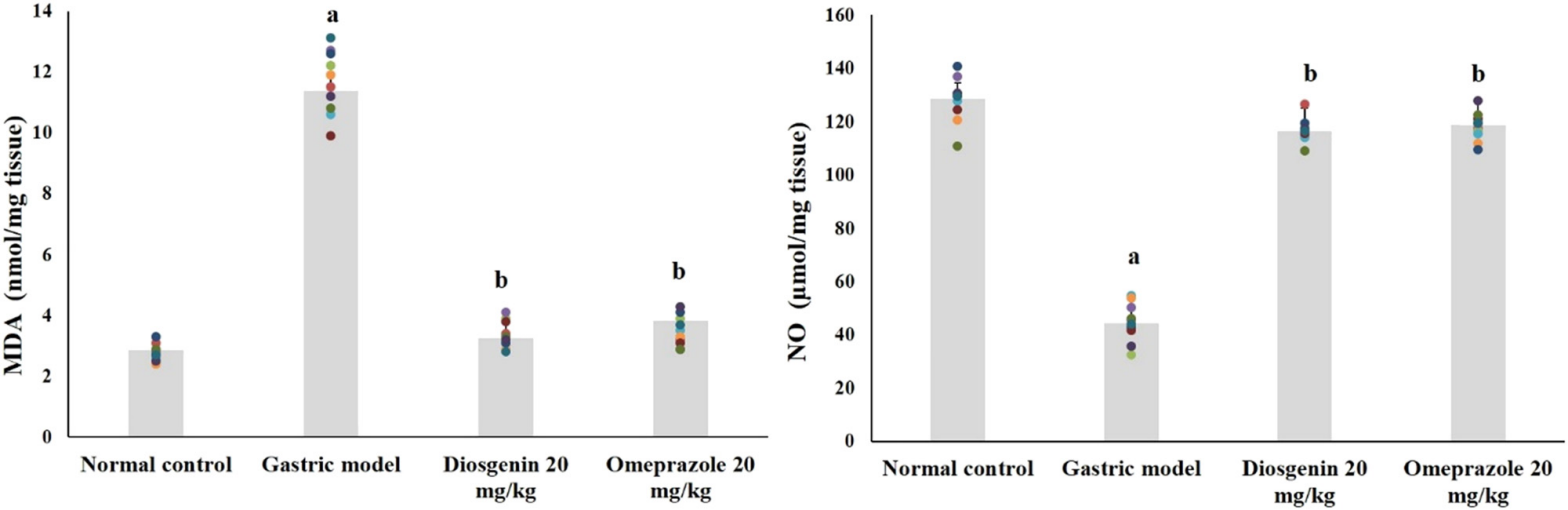
Diosgenin prevents lipid peroxidation and nitric oxide in gastric ulcer-induced rats. Values are expressed as mean ± SEM of lipid peroxidation and nitric oxide levels in rats (*n* = 10). *p*(a) < 0.05 compared to the normal control. *p*(b) < 0.05 compared to the gastric ulcer model. MDA = malondialdehyde; NO = nitric oxide.

Lipid peroxidation indicator MDA and NO levels in the normal and experimental rats are shown in Figure 3. HCl/ethanol-induced gastric model rats showed elevated MDA for about fourfolds from the normal group and suppressed NO by 66% (*p* < 0.05). Instead, intragastric administration of diosgenin decreased MDA and elevated NO (*p* < 0.05) against the model group, similar to the positive control group.

### 3.3 Effects of diosgenin on IL-1β, IL-6, and TNF-α levels

**Figure 4:**
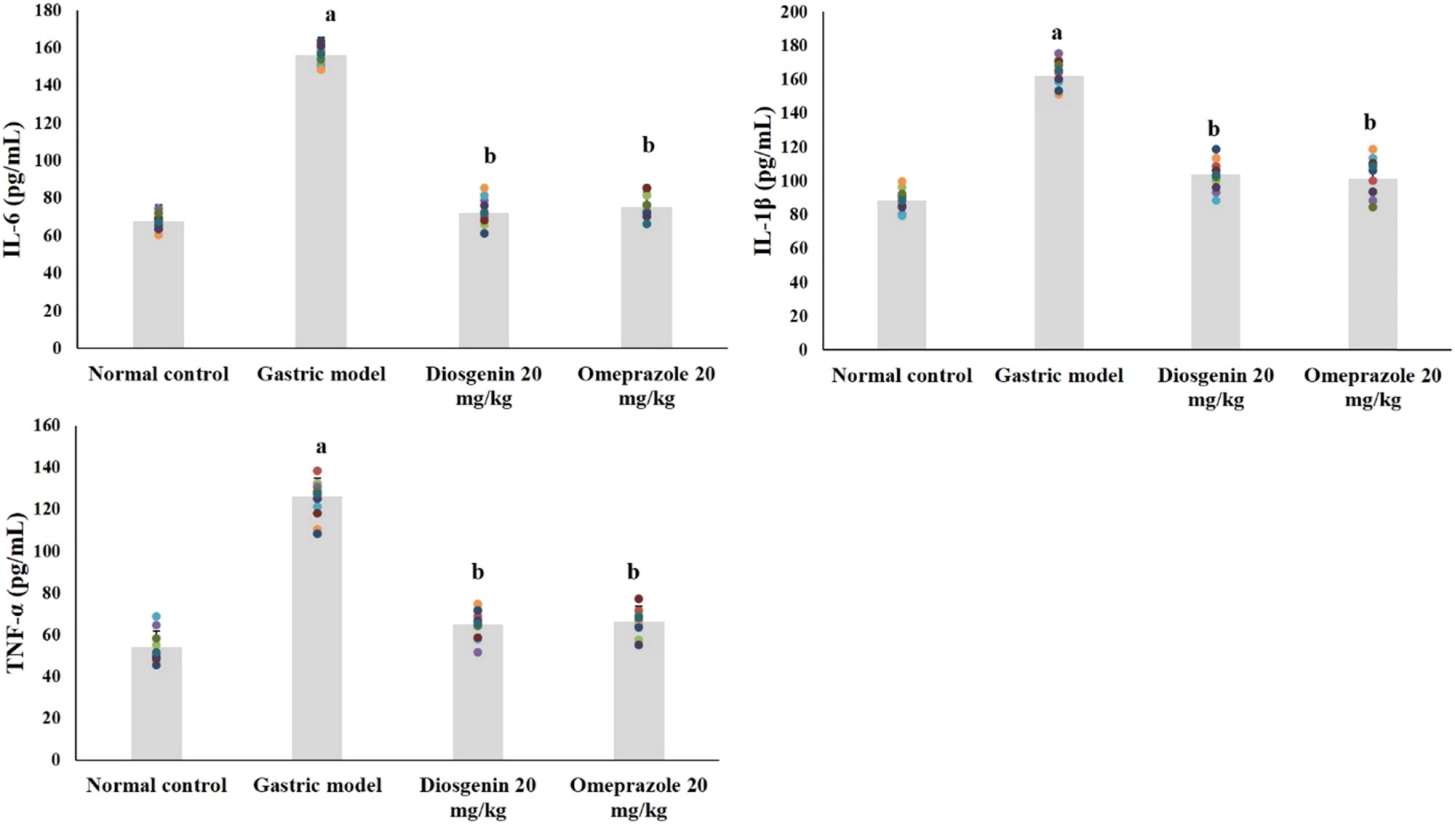
Diosgenin prevents pro-inflammatory cytokines (TNF-α, IL-1β, and IL-6) in gastric ulcer-induced rats. Values are expressed as mean ± SEM of pro-inflammatory cytokines TNF-α, IL-1β, and IL-6 in rats (*n* = 10). *p*(a) < 0.05 compared to the normal control. *p*(b) < 0.05 compared to the gastric ulcer model.

The levels of pro-inflammatory cytokines in normal and experimental rats are shown in Figure 4. Compared to the normal group, IL-1β, IL-6, and TNF-α were significantly elevated due to HCl/ethanol in the model group (*p* < 0.05). Remarkably, IL-1β, IL-6, and TNF-α were reduced (*p* < 0.05) due to intragastric administration of diosgenin from the HCl/ethanol-induced model group. Omeprazole administration exerted similar results as diosgenin.

### 3.4 Effects of diosgenin on MPO activities

**Figure 5:**
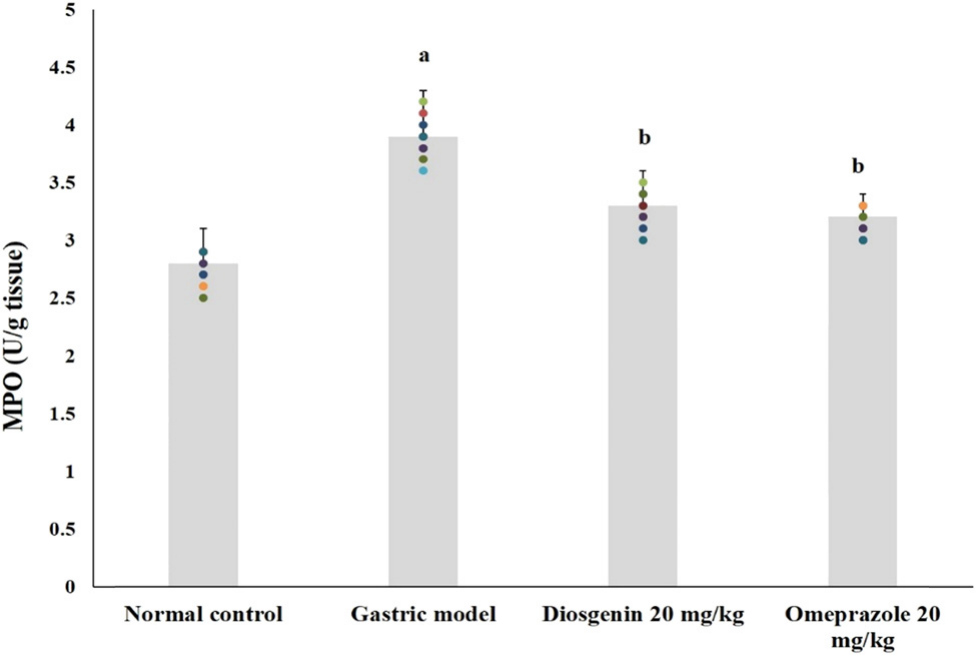
Effects of diosgenin on MPO activities in rat gastric tissue. Values are expressed as mean ± SEM of MPO activities in rats (*n* = 10). *p*(a) < 0.05 compared to the normal control. *p*(b) < 0.05 compared to the gastric ulcer model.

MPO activities of the normal and experimental rats are shown in Figure 5. The activities of MPO in HCl/ethanol-induced model rats were high (*p* < 0.05) up to 40% from the normal group. Compared to the model group, intragastric administration of diosgenin significantly reduced MPO activities (*p* < 0.05) similar to the positive control group.

### 3.5 Effects of diosgenin on histopathological changes

**Figure 6:**
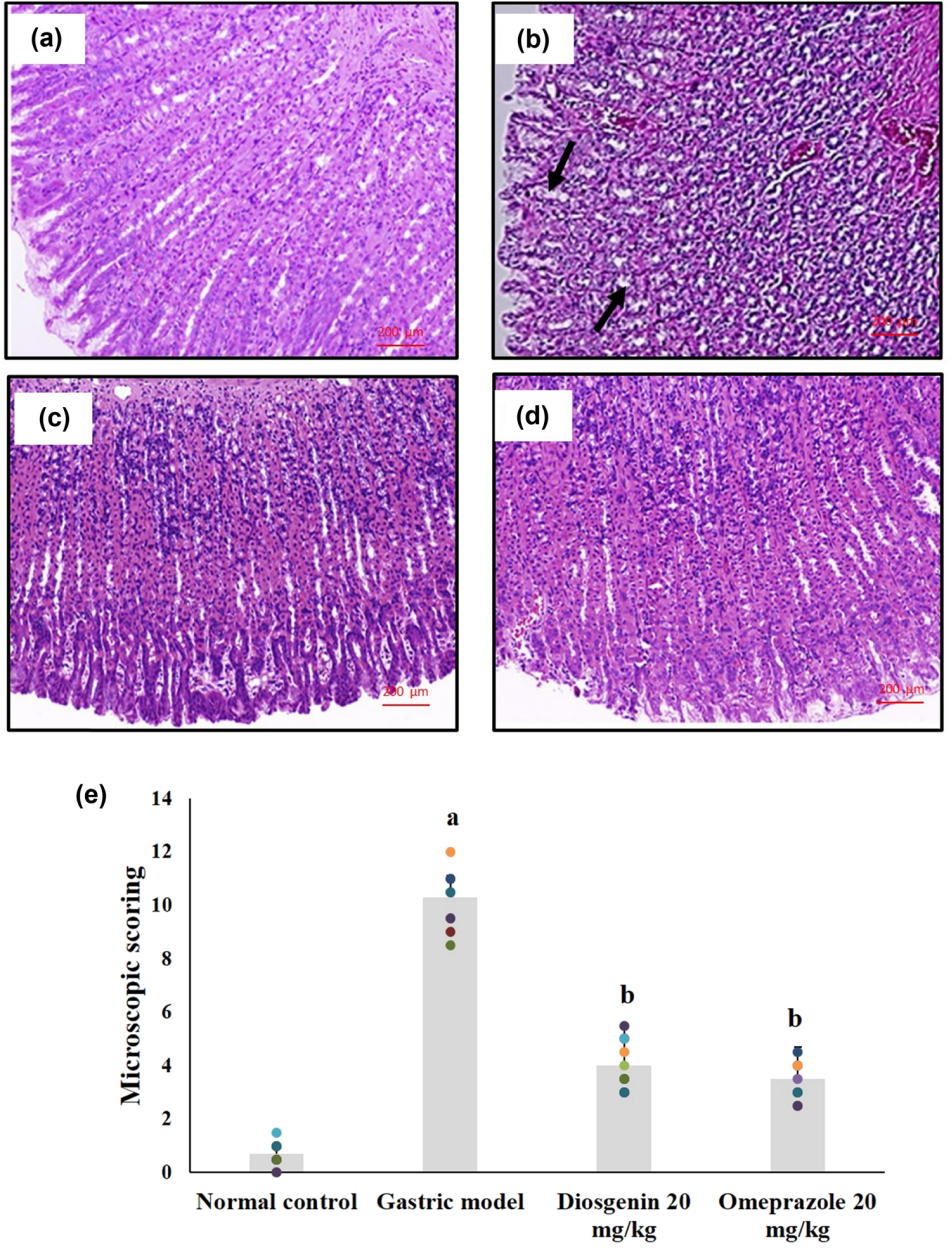
Histopathological changes due to HCl/ethanol-induced gastric mucosal injury and the protective effects of diosgenin. (a) Normal control; (b) gastric model with prominent mucosal injury; (c) diosgenin 20 mg/kg treated gastric tissue with mild mucosal injury; and (d) omeprazole 20 mg/kg treated gastric tissue with very mild mucosal injury. Magnification at 100×. (e) Histopathological scoring of gastric mucosal injury. *p*(a) < 0.05 compared to the normal control. *p*(b) < 0.05 compared to the gastric ulcer model.

Histopathological observations of the normal and experimental rats are shown in Figure 6. The histopathology of the HCl/ethanol-induced gastric injury model showed signs of epithelial cell loss, submucosal edema, hemorrhage, and infiltration of inflammatory cells, indicating mucosal injury compared to the normal rats, which showed normal arrangements and glandular structure of the gastric mucosa. The histopathological scores indicate the mucosal damage caused by HCl/ethanol and the protective measure by diosgenin. Diosgenin administration prevented the changes in rat gastric mucosa by reducing the damage caused by HCl/ethanol, which was comparable to the results of the omeprazole-treated group.

### 3.6 Effects of diosgenin on NF-κβ activation

**Figure 7:**
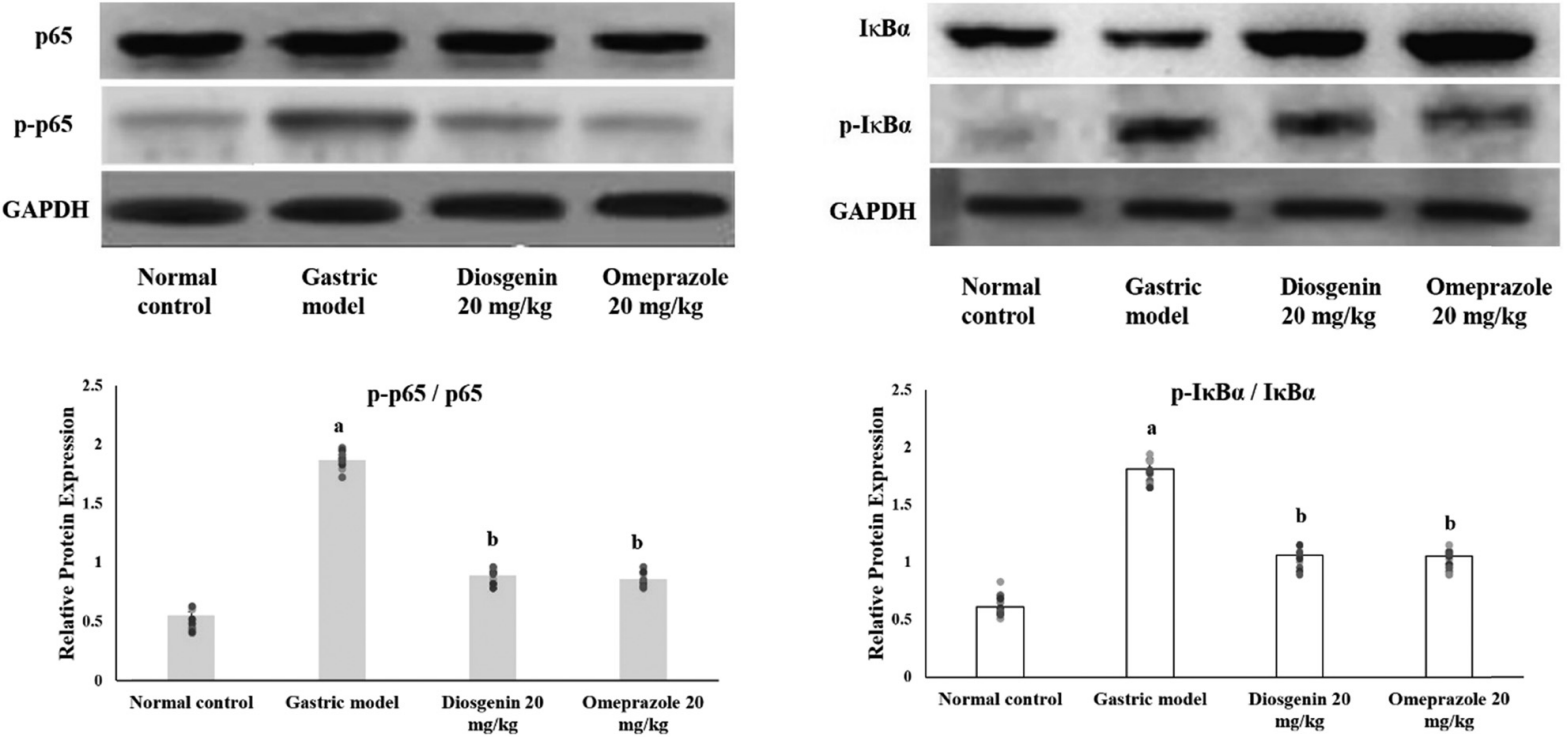
Western blot analysis on NF-κβ in HCl/ethanol-induced gastric mucosal injury and the protective effects of diosgenin. Phosphorylation ratio of p65 NF-κβ and IκBα was quantified and displayed as bar graphs. The bar graphs are the demonstration of three independent results of the western blot analysis, expressed as mean ± SEM. *p*(a) < 0.05 compared to the normal control. *p*(b) < 0.05 compared to the gastric ulcer model. GAPDH was used as the internal standard.

Western blot analysis on the HCl/ethanol-induced gastric mucosal injury-related NF-κβ pathway provided evidence that NF-κβ was activated in the gastric model group by phosphorylation of the subunits as demonstrated by the ratio of phosphorylation in Figure 7. HCl/ethanol administration induced the expression of phosphorylated p65 NF-κβ and Iκβα in the gastric model group compared to the normal control group. Diosgenin significantly suppressed the expressions of phosphorylated Iκβα and p65 NF-κβ compared to the gastric model group.

## 4 Discussion

The present study demonstrated gastroprotective and anti-inflammatory effects of diosgenin against gastric mucosal damage due to HCl/ethanol in rats for the first time. Similar to the previous studies, HCl/ethanol administration caused severe gastric mucosal damage that was observed through gastric ulcer index and histopathological changes with signs of epithelial cell loss, submucosal edema, and hemorrhage [23,24]. Intragastric administration of diosgenin significantly reversed the HCl/ethanol-induced gastric mucosal damage as demonstrated in the results. The gastroprotective effects of diosgenin have similar findings with other bioactive compounds tested in past studies against chemically induced gastric injury in animal models [23,24,25,26,27]. The gastroprotective results of the reported bioactive compounds are associated with their antioxidant and anti-inflammatory effects. In this study, diosgenin was able to increase antioxidant GSH and enzymatic CAT, GPx, and SOD in HCl/ethanol-induced gastric mucosal damage in a similar style to the reference drug omeprazole. Antioxidant enzymes and GSH are regulators of ROS and other free radicals by maintaining the equilibrium of concentration of free radicals formed and scavenged to prevent oxidative stress [8]. Lipid peroxidation is a process of oxidation of lipids forming the cellular membrane by excessive free radicals formed during oxidative stress conditions [3]. The gastric injury model rats were in a state of oxidative stress due to the increased levels of MDA, suppressed NO levels, and diminished GSH, GPx, SOD, and CAT activities. Oxidative stress often leads to the pathophysiology of many diseases including gastric mucosal injury and ulcer [26]. The levels of MDA were incisively reduced, and the NO levels were replenished by intragastric administration of diosgenin. HCl/ethanol administration triggers endogenous inhibitors of nitric oxide synthase (NOS) enzyme, causing the suppression of NO production. It has been reported that the increased NO levels help the healing of gastric mucosal injury [8]. Therefore, the antioxidant activity of diosgenin was prevailing in the results through prevention of oxidative stress and prevention of the inhibition of NOS.

Anti-inflammatory effects of diosgenin were proven in the results of the ELISA assay on IL-1β, IL-6, and TNF-α, where diosgenin suppressed activities of pro-inflammatory cytokines. The gastric injury model rats were having severe inflammation at the site of gastric mucosal injury evidenced by the ELISA assay results on pro-inflammatory cytokines and the histopathological findings of inflammatory cells infiltration. TNF-α and IL-6 are predominantly involved in the inflammatory reaction in gastric mucosal injury by recruiting other inflammatory mediators to the site of injury and also triggers oxidative stress [27]. Accumulation of these pro-inflammatory cytokines increases the damage to surrounding gastric cells. Suppressing these pro-inflammatory cytokines could be a potent therapeutic approach to cure gastric mucosal damage. The activity of MPO is directly related to the inflammatory process during gastric mucosal damage. Increased MPO activity is an indicator for high neutrophil infiltration to the site of gastric mucosal damage since MPOs are released by stimulated neutrophils [28]. The MPO activity of the HCl/ethanol-initiated gastric mucosal damage model group indicated the high rate of neutrophil infiltration. Diosgenin administration significantly reduced the MPO activities. NF-κβ is an important factor in triggering inflammatory reactions, which has control over genes of inflammatory mediators. Translocation of NF-κβ into the nucleus initiates the transcription of TNF-α, IL-6, and other inflammatory markers. Previous studies on the involvement of NF-κβ in gastric mucosal injuries make it an important target for the therapeutic effect of gastric injuries [29,30]. Diosgenin significantly prevented the translocation of NF-κβ as evidenced by the western blot results. Therefore, the anti-inflammatory role of diosgenin and gastroprotective effects might be due to the downregulation of NF-κβ and inhibition of MPO activities through the prevention of inflammatory cells infiltration.

## 5 Conclusion

Overall, this study finds that diosgenin has significant gastroprotective and anti-inflammatory roles against HCl/ethanol-induced gastric mucosal damage in rats. The antioxidant effects of diosgenin significantly attenuated oxidative stress by improving the antioxidant enzyme activities, reducing MDA and NO formations. Moreover, the anti-inflammatory role of diosgenin was exerted by downregulation of NF-κβ and MPO in the stomach of HCl/ethanol-induced rats comparable to the action of the commercial drug omeprazole. Therefore, diosgenin can be promoted as an important food ingredient to prevent gastric injury. Further researches are needed to determine the safe dose and toxicity levels of the compound before be included as an essential food additive.
